# Protective role of the alpha-1-antitrypsin in intervertebral disc degeneration

**DOI:** 10.1186/s13018-021-02668-z

**Published:** 2021-08-20

**Authors:** Weikun Liu, Yanfu Wang

**Affiliations:** 1 Department of Orthopedics, People’s Hospital of Dongxihu District, Wuhan, Hubei People’s Republic of China; 2 Department of Rehabilitation Medicine, The Central Hospital of Wuhan, Tongji Medical College, Huazhong University of Science and Technology, Wuhan, Hubei People’s Republic of China

**Keywords:** Intervertebral disc degeneration, Alpha-1 antitrypsin, Apoptosis, Extracellular matrix degradation, WNT/β-catenin signaling

## Abstract

**Background:**

Intervertebral disc degeneration is a complex disease with high prevalence. It suggests that cell death, senescence, and extracellular matrix degradation are involved in the pathogenesis. Alpha-1 antitrypsin (AAT), a serine protease inhibitor, was previously correlated with inflammation-related diseases. However, its function on intervertebral disc degeneration remains unclear.

**Methods:**

A latex-enhanced immunoturbidimetric assay measured the serum level of AAT. Real-time polymerase chain reaction (RT-qPCR) and western blot were used to testify the expression of RNA and proteins related to cell apoptosis and the Wnt/β-catenin pathway. The animal model for intervertebral disc degeneration was built by disc puncture. The degeneration grades were analyzed by safranin o staining.

**Results:**

We showed that alpha-1 antitrypsin could ameliorate intervertebral disc degeneration in vitro and in vivo. We also found that the serum alpha-1 antitrypsin level in Intervertebral disc degeneration patients is negative related to the severity of intervertebral disc degeneration. Moreover, alpha-1 antitrypsin was also showed to suppress tumor necrosis factor-alpha (TNF-α) induced WNT/β-catenin signaling pathway activation in human nucleus pulposus cells.

**Conclusions:**

Our study provides evidence for AAT to serve as a potential therapeutic reagent for the treatment of intervertebral disc degeneration.

## Introduction

Low back pain (LBP) affects millions of people worldwide and leads to an enormous socioeconomic burden [1, 2]. In the USA, spinal fusion surgery, with $12.8 billion costs in 2011, was responsible for the highest aggregate hospital costs of any surgical procedure [3]. It was estimated that more than 480,000 lumbar discectomy cases occurring annually in the USA [4]. Although many efforts have been spent on preventing and treating this disorder, the underlying etiology and pathogenesis mechanism remain incompletely understood. It is also urgent to explore a potential therapeutic method for the treatment.

Intervertebral disc (IVD) degeneration is a leading contributor to LBP. IVD are fibrocartilaginous cushions between spinal vertebrae, composed of an annulus fibrosus and a nucleus pulposus. The primary mechanism of IVD degeneration consists of the degradation of the extracellular matrix (ECM) and the degeneration of nucleus pulposus (NP). NP cells are the primary cell type in the NP; they play an essential role in tissue homeostasis inside the NP tissue. NP cells are rounded, “chondrocyte-like” morphology; however, it has distinct phenotype and characteristics compared with chondrocytes [5].

α1-antitrypsin (AAT) was discovered in the mid-1950s but only came to prominence in 1963. It was identified as a potent serine protease inhibitor [6], with the highest affinity for neutrophil elastase (NE) [7]. AAT was produced by hepatocytes and secreted in the blood. It plays a central role in limiting local and systemic inflammation, and AAT concentrations rise ~ 4-fold during infection and inflammation, remaining elevated up to 7 days, suggesting a function in host protection. Researchers have found that AAT is endowed with anti-inflammatory, analgesic, and chondroprotective properties partially interrelated. AAT has been shown to play a role in several conditions, like inflamed pancreatic islets, arthritis, autoimmune encephalomyelitis, and autoimmune encephalomyelitis [8, 9].

As far as we know, this is the first study that explored the relationship between AAT concentrations and disease progression in IVD degeneration. Moreover, our results indicated that AAT contributes to IVD degeneration by alleviating NP cell apoptosis by regulating the Wnt/β-catenin pathway. This study implied that AAT might serve as a novel serum marker for disease prediction and a potential therapeutic reagent for the treatment of IVD degeneration.

## Results

### AAT levels in IVD degeneration patients and controls

**Fig. 1:**
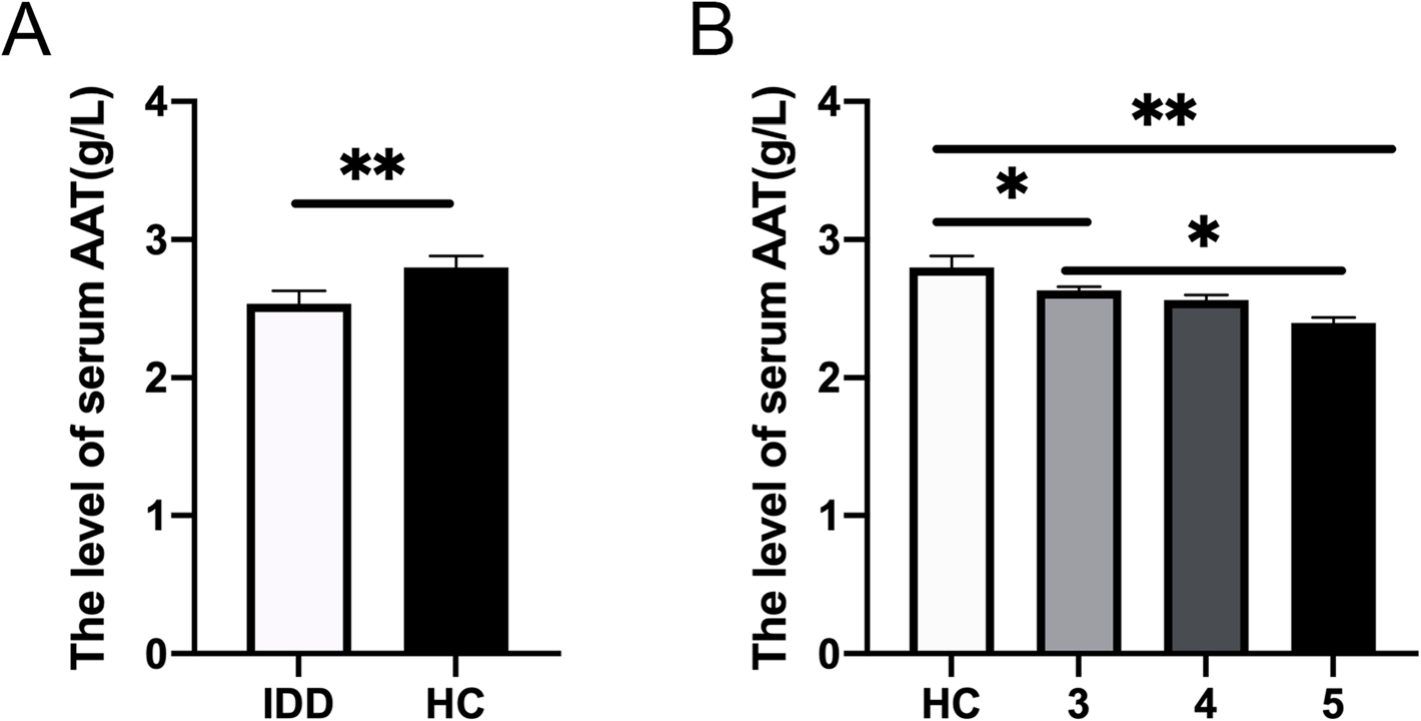
**A** Comparison of serum levels of AAT between disc degeneration patients and control participants. **B** The serum level in disc degeneration patients with different disease severity, classified by Pfirmann grade. HC = healthy control. *N* = 15 for each group. (*p < 0.05, **p < 0.005, ***p < 0.001)

Serum AAT concentrations were found to be significantly lower in symptomatic IVD degeneration patients (2.53 ± 0.089 g/L), compared with asymptomatic healthy individuals (2.797 ± 0.081 g/L) (Fig. 1A). IVD degeneration severity was determined by participant’s MRI scans, based on the Pfirrmann grading system [10]. It was found that serum AAT concentrations were highest in participants without disc degeneration. The serum AAT level was significantly higher in patients with grade III degeneration than those with grade IV or V degeneration (Fig. 1B). Overall, our results showed that serum AAT levels were negatively correlated with the severity of IVD degeneration.

### AAT protects TNF-α-induced disc degeneration

**Fig. 2:**
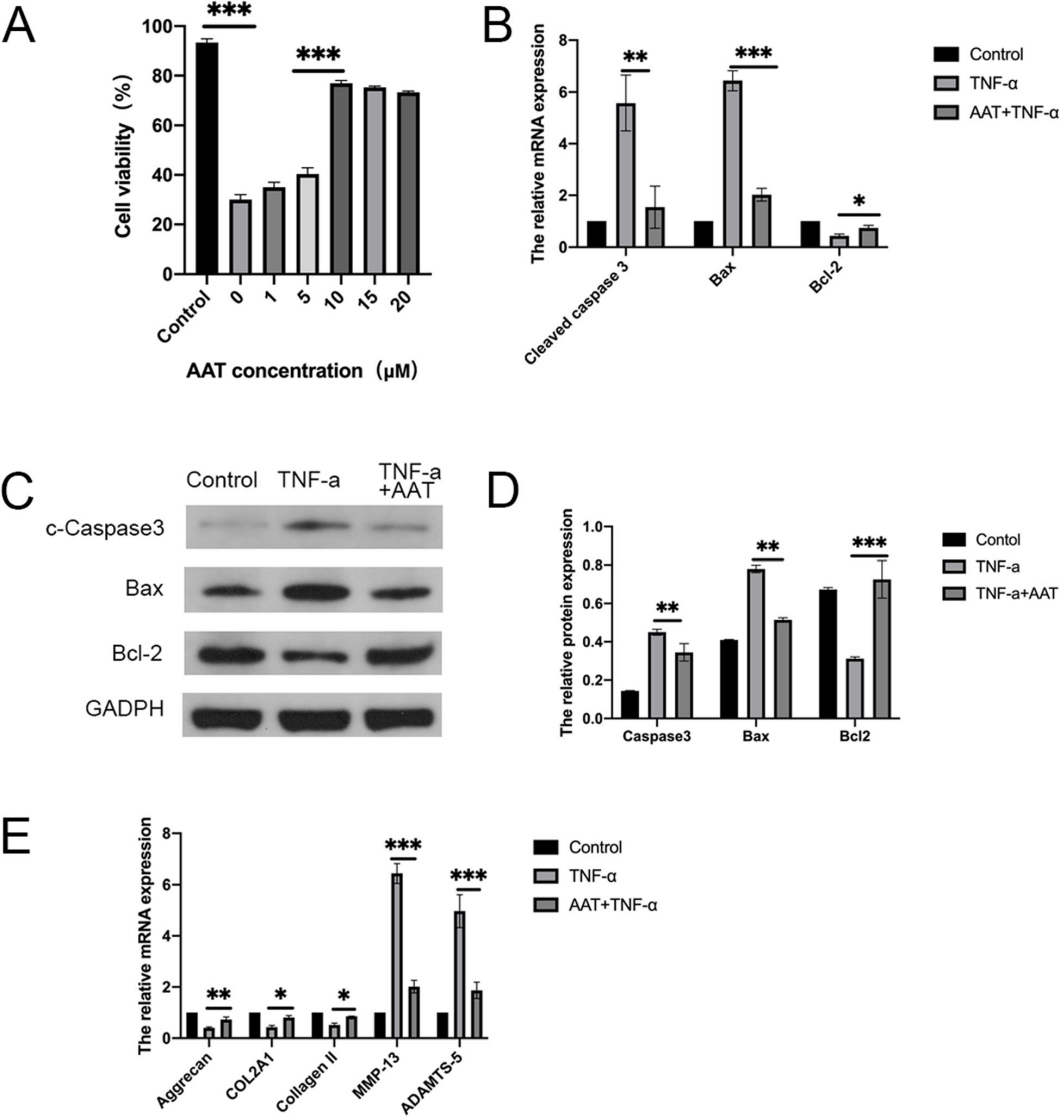
**A** Cell viability of N.P. cells after treatment with 25 ng/mL TNF-α or not, and then incubation with 1, 5, 10, 15, or 20 μM AAT for 48 h. **B** RT-qPCR was used for analyzing the RNA expression of cleaved caspase 3, Bax, and Bcl-2 in human N.P. cells. **C**, **D** Western blot was used for analyzing the expression of cleaved caspase 3, Bax, and Bcl-2 in human N.P. cells at the protein level. **E** The expression of ECM synthesis genes (Aggrecan, COL2A1, Collagen II) and matrix-degrading enzymes (MMP-13, ADAMTS-5) was detected by RT-qPCR. All data were based on at least three independent experiments. (*P < 0.05, **P < 0.01, ***P < 0.001)

To determine the effect of AAT on the relative growth rate of TNF-α-treated NP cells, a CCK-8 assay was performed after 48 h of culture under basal conditions in the presence of 1, 5, 10, 15, or 20 μM AAT after treating by 25 ng/mL TNF-α for 48 h or TNF-α alone. Results indicated that AAT effectively improved NP cells viability at 10 μM concentrations (Fig. 2A). Therefore, we used an AAT concentration of 10 μM in the following experiments. RT-qPCR showed Bcl-2 expression decreased, but Bax expression increased in the TNF-α group compared with the AAT + TNF-α group (Fig. 2B). Western blots revealed the expression of cleaved caspase-3 and Bax increased, but Bcl-2 decreased significantly in AAT-treated group compared with TNF-α alone group at the protein level (Fig. 2C, D). We next explored the effects of AAT on ECM. Our results showed that AAT could upregulate the expression of ECM synthesis genes: Aggrecan, COL2A1, and Collagen II, and significantly downregulate the expression of matrix-degrading enzymes: MMP-13, ADAMTS-5 (Fig. 2E). These findings indicated that AAT has a protective effect on the degenerated NP cells.

### AAT suppressing WNT/β-catenin signaling pathway in NP cells

**Fig. 3:**
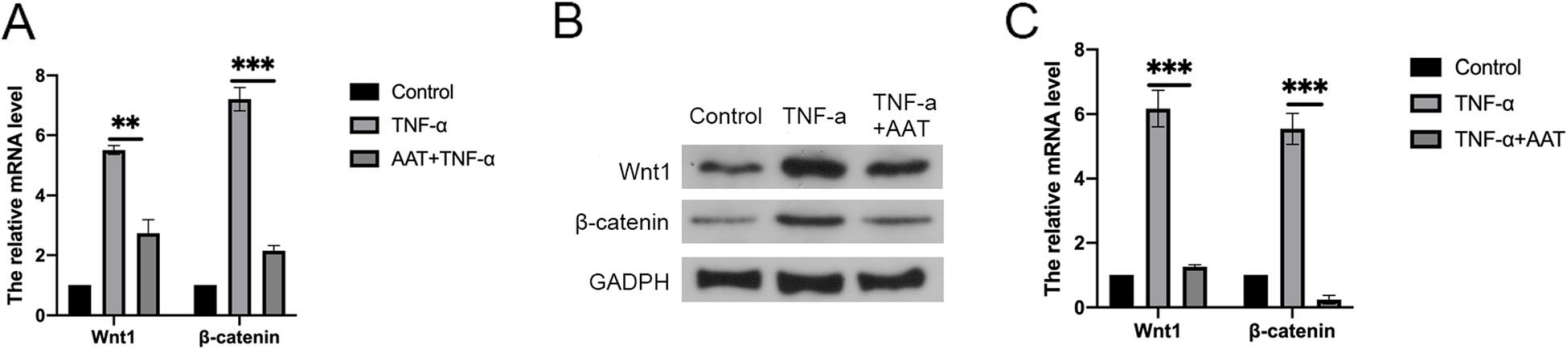
**A** The expression levels of Wnt1 and β-catenin in the N.P. cells detected by RT-qPCR. **B**, **C** The protein expressions of Wnt1 and β-catenin in the NP tissues were detected by Western blot. GAPDH was used as internal controls. *P < 0.05, **P < 0.05, ***P < 0.001 compared to TNF-α group

The gene expression levels of Wnt1 and β-catenin increased significantly in TNF-α treated NP cells compared with untreated control NP cells. AAT treatment decreased the expression of both Wnt1 and β-catenin (Fig. 3A). The western blot analysis showed that TNF-α increased the level of Wnt1 and β-catenin. AAT treatment significantly inhibited the expression of these two proteins (Fig. 3B, C).

### AAT ameliorates IVD degeneration in vivo

**Fig. 4:**
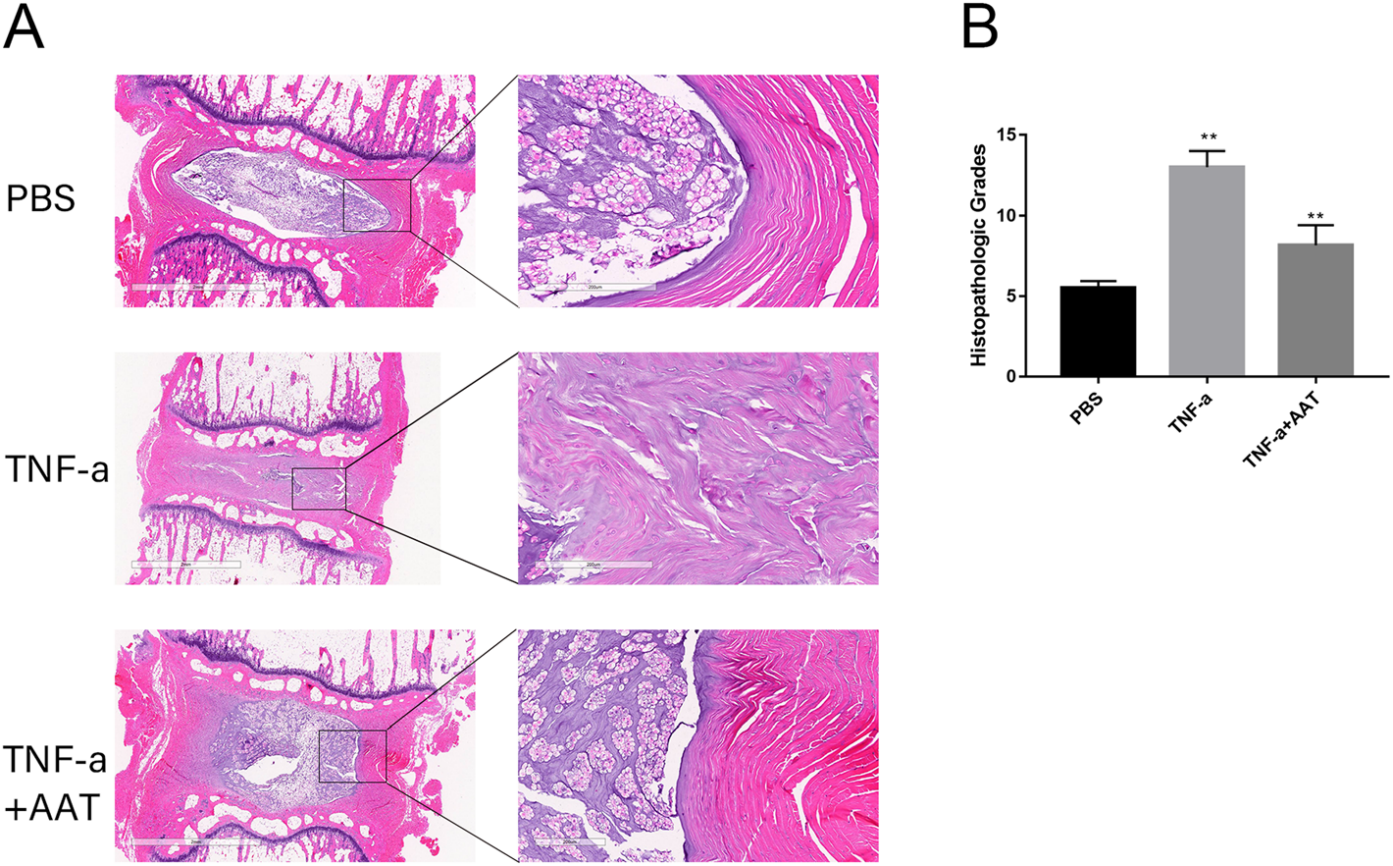
The rat tail discs were injected with PBS, TNF-α (25 ng/mL), and TNF-α (25 ng/mL + ATT (10 μM), maintained for 1 month. **A** Whole tail discs were observed using safranine O staining. **B** Safranine O staining images according to histological grading scale *p < 0.05 versus PBS. *p < 0.05 versus ATT

To further investigate the therapeutic effects of ATT on degenerated IVD degeneration, an animal model of IVD degeneration was used in our experiment. The IVD specimens from the above animal models were subjected to histopathological analysis and scores. As shown in Fig. 4A, the PBS treated discs showed high glycosaminoglycan content. The oval-shaped NP occupied a large volume of the disc (> 50%) in the midsagittal cross-section. In TNF-α-injected groups, the disc height decreased significantly. The safranine O staining demonstrated the evident cells loss, NP/AF boundary disappeared and increased fibrillation in the NP area. However, in ATT administrated groups, the degenerative changes were alleviated compared with TNF-α-injected groups. There was still some loose NP tissue (> 25%) with stellar-shaped cells and glycosaminoglycan content detected by safranine O staining. Moreover, the NP/AF boundary was still clear, and the height of the discs was moderate. We next evaluated the histopathological scores using a grading scale described previously [11] (Fig. 4B). These results indicated that ATT could ameliorate the TNF-α induced IVDD in vivo

## Materials and methods

### Human samples

Normal human NP cells were obtained from patients with idiopathic scoliosis (*n* = 5; average age, 26.8 years; range, 18–35 years). Degenerative NP specimens were obtained from patients who were suffering from intervertebral disc degeneration caused by different conditions, including lumbar spinal stenosis, spondylolisthesis, and lumbar disc herniation.

### Serum analysis

AAT (g/liter) concentrations were determined by latex-enhanced immunoturbidimetric assay, a robust assay with perfectly comparable principles to those of nephelometry. α1-AT phenotypes were determined by isoelectric focusing in polyacrylamide gels at pH values between 3.5 and 5.0, using genomic DNA extracted from peripheral blood [12].

### Isolation and culture of human IVD tissues

This procedure was conducted as previously reported and performed under sterile conditions [13]. Human IVD tissues were derived from patients undergoing traumatic lumbar surgery. Briefly, the annulus fibrous and endplates were meticulously removed, and explants were cultured in DMEM/F-12 culture medium (HyClone, Thermo Co., USA) supplemented with 10% fetal bovine serum (FBS, Gibco, USA), 1% 100 U/mL penicillin and 100 mg/mL streptomycin (HyClone, USA). The indicated culture groups were stimulated with TNF-α at 24 h after isolation as previously reported [14].

### Rat model establishment

The rat IVD degeneration model was established as previously reported [15]. The area between the eighth and ninth coccygeal vertebrae (Co8–Co9) in rats was punctured using a 20-gauge needle. To make sure that the needle could not be punctured too deep, the length of the needle was decided according to the annulus fibrosus and the nucleus pulposus dimensions, which were measured in the preliminary experiment and found to be about 4 mm. All the needles were kept in the disc for 1 min. AAT was diluted with normal saline to achieve a final AAT concentration of 10 mg/ml. After surgery, the AAT solution was immediately injected intraperitoneally to deliver a dose of 50 mg/kg/day until the rats were killed. Daily monitoring of the rats was carried out to ensure their well-being, and all animals were allowed free unrestricted weight-bearing and activity.

### Cell viability assay

NP cells viability were measured using a Cell Counting Kit (CCK-8, Dojindo, Japan) according to the manufacturer’s instructions. NP cells were seeded in 96-well culture plates at a density of 2 × 10^3^ cells per well. Ten microliters of CCK-8 solution was applied at specific time points; then, cells were incubated in the dark for 2 h at 37 °C. Cell viability was assessed through absorbance detection at 450 nm using a spectrophotometer (ELx808 Absorbance Microplate Reader, Bio-Tek, USA).

### Real-time PCR

Total RNA was extracted from human NP cells and tissues using TRIzol reagent (Ambion, Foster City, CA, USA) according to the manufacturer’s instructions. The primers used for qRT-PCR were listed in Table 1. Total RNA was reverse transcribed using PrimeScript RT Master Mix (Takara Bio, Shiga, Japan) according to the manufacturer’s instructions. qRT-PCR was performed using the One-Step SYBR PrimeScript RT-PCR Kit (Takara Bio) to quantify the RNA or mRNA expression levels of Bax, Bcl-2, Cleared Caspase-3, COL2A1, MMP-13, ADAMTS-5, type II collagen, aggrecan, Wnt1, and β-catenin. Target mRNA expression levels were normalized against GAPDH. The relative expression levels were computed using the 2−ΔΔCt method.

**Table 1:** Sequences of primers used for quantitative real-time PCR

Gene	Forward (5–3′)	Reverse (5–3′)
Bax	AACTGGGGTCGATTGTG	GATCCAAGGCTCTAGGTGGTC
Bcl-2	TTGAGTTCGGTGGGGTCATG	GATCCAGGTGTGCAGATGCC
Cleared Caspase-3	TATGGAATTGATGGATAGT	CTGAAGAAACTAGTTAGTT
Aggrecan	TCCAAACCAACCCGACAAT	TCTCATAGCGATCTTTCTTCTGC
COL2A1	ACGCTCAAGTCGCTGAACAA	TCAATCCAGTAGTCTCCGCTCT
Collagen II	GGGAATGTCCTCTGCGATGAC	GAAGGGGATCTCGGGGTTG
MMP-13	CCGAGGAGAAACAATGATCT	GCCTGTATCCTCAAAGTGAA
ADAMTS-5	CGACAAGAGTCTGGAGGTGAG	CGTGAGCCACAGTGAAAGC
Wnt1	GAATCGCCGCTGGAACTGTC	GCGGAGGTGATAGCGAAGATAAACG
β-catenin	CAAGTGGGTGGTATAGAGG	AGTCCATAGTGAAGGCGAAC
GAPDH	TCAAGAAGGTGGTGAAGCAGG	TCAAAGGTGGAGGAGTGGGT

### Western blot analysis

Proteins were extracted from NP cells using RIPA lysis buffer. Western blotting was carried out as described previously [16] with antibodies against the following proteins: cleaved caspase-3, B cell lymphoma-2(Bcl-2), B cell lymphoma-2 associated X (Bax), Cleared Caspase-3, Wnt1, and β-catenin. After initial incubation, the membrane was cultured with horseradish peroxidase (HRP)-conjugated goat anti-rabbit secondary antibody (Boster, Wuhan, China). The grey value ratio of the target band to the reference band reflected the relative protein levels with GAPDH as the internal reference protein.

### Histopathologic analysis

The rats were killed by an intraperitoneal overdosage injection of 10% chloral hydrate, and the tails were next harvested. The specimens were decalcified and fixed in formaldehyde, dehydrated, and embedded in paraffin. The tissues were cut into 5-μm sections. Slides of each disc were stained with safranine O. The cellularity and morphology of nucleus pulposus and annulus fibrosus were examined by another group of experienced histology researchers in a blinded manner using a microscope and evaluated by using a grading scale, as described previously [11]. The histologic score was 5 for the normal disc, 6–11 for moderately degenerated disc and 12–14 for the severely degenerated disc.

### Statistical analysis

Total data acquisition was conducted in a blinded manner. For comparisons of various treatment groups, the unpaired Mann-Whitney *t* test paired Student’s *t* test, and 1-way or 2-way ANOVA (when appropriate) were performed. For ANOVA, Bonferroni post hoc analysis was used to compare treatment groups. All statistical analyses were performed with GraphPad Prism software (version 7.0; GraphPad Inc., La Jolla, CA, USA). The results are presented as the mean ± standard deviation (S.D.) based on three independent experiments. The differences between groups were analyzed using the Student’s *t* test or one-way analysis of variance (ANOVA).

## Discussion

Low back pain (LBP) is a common pathological condition, resulting in immense healthcare and social-economic burdens [17]. It has been widely acknowledged that intervertebral disc degeneration (IVD) is a major cause of LBP [18]. IVD consists of three conspicuous, including the inner gelatinous nucleus pulposus (N.P.), the outer annulus fibrosus (A.F.) and lower thin cartilages endplates (CEP) [19]. The degeneration of inner N.P. was found to be a significant important mechanism for IVD degeneration. N.P. is the central composite of IVD, rich in ECM. The ECM of N.P. is composed predominantly of type II collagen, proteoglycan aggregate, and aggrecan. Other numerous fewer components include biglycan, cartilage oligomeric matrix protein, decorin, and fibronectin [20]. Until now, the treatments for IVDD are relied on surgeries, like discectomy or spinal fusion procedures. Given the insufficient regenerative capacity for N.P. tissues, it is necessary to explore the biological method for alleviation or inhibiting the pathogenesis for IVD degeneration.

α1-antitrypsin (AAT) is a water-soluble and tissue diffusible circulating serine protease inhibitor. Previous studies showed that AAT could prevent tissue damage and inflammation via inactivating the serine protease elastase released by neutrophils [21]. The AAT concentration in plasma has previously been shown to increase in inflammation conditions, especially in the acute phase [22]. The level of AAT in a healthy individual is 0.9–2 g/L in plasma [23]. Some studies showed it might multiply 4–5 times in some acute inflammation and infection cases and increased in the third trimester of pregnancy or aging [23] AAT deficiency is one of the most important causes of chronic obstructive pulmonary disease (COPD) worldwide. Apart from respiratory diseases, the deficiency of AAT may also cause systemic vasculitis, panniculitis, type 2 diabetes mellitus, and spontaneous abortions [24]. In our study, we first showed that in IVD degeneration patients, the serum level of AAT decreased significantly compared to healthy individuals. These indicated that AAT presented a protective role for IVD degeneration.

Apoptosis, an essential type of IVD cell death, has been considered to play a crucial role in the process of degeneration [25]. Previously, studies showed that AAT has a direct pro-survival effect in apoptosis-dependent emphysema models [26]. Our data showed that AAT could inhibit the activity of caspase-3, the main executioner caspase involved in apoptosis, in the TNF-α induced NP cells degeneration model. Karina et al. showed intracytoplasmic uptake of AAT by lung endothelial cells revealed partial colocalization with caspase 3 [27]. The anti-apoptosis capacity of ATT providing the underlying mechanisms for its treatment potential. It was also suggested that AAT could inhibit the production of proinflammation cytokines, including IL-1beta and TNF-α, and promoting the release of anti-inflammation cytokines, like IL-10 [28, 29]. During the process of IVDD, the inflammation cascade accelerates the pathogenesis. Given the capacity of ATT, it should also be possible that ATT may alleviate the generation of proinflammation cytokines. Further studies need to be conducted to show these hypotheses.

These protective effects for AAT were found to be associated with Wnt/β-catenin signaling pathways in NP cells. This signal pathway plays a harmful impact on the health of IVD. Wnt proteins are important regulatory factors for IVD. The activation of Wnt/β-catenin signaling could suppress proliferation and induce senescence in NP cells [30]. Tumor necrosis factor-α (TNF-α) was found highly expressed in degenerative IVD tissues, and it was demonstrated deeply involved in IVDD pathogenesis, including extracellular matrix degeneration, apoptosis, and cell proliferation [31]. Here, we built an in vitro model of TNF-α induced NP degeneration. Seguin et al. reported that TNF-α reduced the synthesis of matrix molecules and upregulated the mRNA expression of MMP-1, -3, and -13 and ADAM-TS4 and ADAM-TS5 [32]. These data indicated that AAT could alleviate TNF-α-induced cell apoptosis and ECM degeneration.

The Wnt/β-catenin signaling pathway is a fundamental and evolutionarily conserved mechanism that directs cell proliferation, polarity, cell fate determination, and tissue homeostasis [33]. The interaction between TNF-α components and Wnt/β-catenin signaling has also been analyzed.

It has been demonstrated that the Wnt/β-catenin pathway plays a significant role in chondrocyte proliferation and hypertrophy [34]. Moreover, the expression of SOX-9 and collagen II are elevated when the Wnt/β-catenin pathway is inhibited. SOX-9 and collagen II are protective genes against chondrogenesis and disc degeneration [35, 36]. Akihiko et al. founded the activation of Wnt/β-catenin signaling can induce both NP cells degeneration and TNF-α activation, forming a positive feedback loop in NP cells [37]. When Wnt/β-catenin signaling activates, NP cells’ proliferation was suppressed, but the cellular senescence was accelerated during IVD degeneration [38]. Here, we showed ATT could be a reagent for stopping this positive feedback system in TNF-α stimulated NP cells. More experiments should be performed to understand the role of the Wnt/β-catenin signaling pathway in NP cells.

In conclusion, the current study represents the first demonstration of the protective role of AAT in IVD degeneration, showed the possible mechanism by which ATT is targeting TNF-α induced Wnt signal pathway activation.

## Data Availability

The data that support the findings of this study are available from the corresponding author on reasonable request.

## Abbreviations

AAT: Alpha-1 antitrypsin
TNF-α: Tumor necrosis factor-alpha
IVD: Intervertebral disc
ECM: Extracellular matrix
N.P.: Nucleus pulposus
NE: Neutrophil elastase
RT-qPCR: Real-time polymerase chain reaction
